# Individual Differences in Emotion Dysregulation and Social Anxiety Discriminate between High vs. Low Quality of Life in Patients with Mild Psoriasis

**DOI:** 10.3390/jpm11111236

**Published:** 2021-11-21

**Authors:** Edita Fino, Paolo Maria Russo, Vera Tengattini, Federico Bardazzi, Annalisa Patrizi, Monica Martoni

**Affiliations:** 1 Department of Experimental, Diagnostic and Specialty Medicine (DIMES), Alma Mater Studiorum Università di Bologna, St. Orsola-Malpighi Hospital, Via Massarenti 9, 40138 Bologna, Italy; p.russo@unibo.it (P.M.R.); monica.martoni@unibo.it (M.M.); 2 Dermatology Unit, Sant’Orsola-Malpighi Hospital, Via Massarenti 9, 40138 Bologna, Italy; vera.tengattini@hotmail.com (V.T.); federico.bardazi@unibo.it (F.B.)

**Keywords:** emotion dysregulation, health-related quality of life, individual differences, social anxiety, psoriasis

## Abstract

A deeper understanding of how health-related quality of life relates to the clinical and individual characteristics of patients is essential for the delivery of patient-centered dermatological care. The current study aimed to examine the role of individual differences in emotion dysregulation and social anxiety in modulating quality of life in psoriatic patients. A total of 130 patients affected by psoriasis were consecutively enrolled in the study as they approached the Dermatology Unit of Sant’Orsola-Malpighi Hospital of Bologna. Clinical information gathered included illness severity, assessed with the Psoriasis Area and Severity Index (PASI) and the Body Surface Area (BSA); illness onset; familiarity; and prescribed treatment. The patient-reported outcome measures were the Dermatology Life Quality Index (DLQI), measuring the patient’s quality of life; the Psoriasis Skin Appearance Bothersomeness scale (PSAB), measuring patient’s perception of illness severity; the Difficulties in Emotion Regulation Scale (DERS), assessing emotion dysregulation traits; and the Social Interaction Anxiety Scale (SIAS), measuring anxiety about social interactions. Patients with moderate-to-severe psoriasis reported significantly lower quality of life compared to mildly affected patients. In addition, of the patients affected by mild psoriasis, those characterized by emotion dysregulation and social anxiety traits showed significantly lower levels of quality of life. Our findings suggest that individual differences in emotion dysregulation and social anxiety contribute to health-related quality of life in addition to illness severity. Therapeutic approaches that combine dermatological care with psychological support, especially focused on emotional regulation skills, may be useful to improve clinical outcomes in patients with psoriasis.

## 1. Introduction

The largest organ of the body, the skin, is also one’s interface with the surrounding environment as it facilitates physical contact, social interaction and communication of emotions [1]. For this reason, chronic skin diseases, especially those involving visible markers like psoriasis which is characterized by lesions arising from an uncontrolled proliferation of skin cells, are commonly associated with significant psychosocial distress [2,3,4]. Despite it being a non-life-threatening condition, living with psoriasis can trigger high levels of anxiety about interacting with others as well as negative emotions [5,6]. In addition to illness severity, the visibility of markers and their location on specific body parts can act as a stressor, as it can be associated with negative reactions from others, resulting in higher feelings of stigmatization [7,8,9]. For instance, lesions located in the head and neck area, as well as on the arms, hands and genitals, are associated with a higher risk of psychosocial distress [8]. Importantly, while the psychosocial burden of disease may vary depending on the type, extension and location of lesions, patients’ perceptions of the illness may affect self-esteem, including perceptions of physical appearance. This has important implications for the relational sphere. In fact, patients with psoriasis are unusually sensitive to and anxious about the judgments and reactions of others, which quite often reflect stigmatizing attitudes and behaviors [7,8,9]. For the aforementioned reasons, it has been recognized that psoriasis can have a profound impact on patient health-related quality of life (HrQoL) [10,11,12,13]. 

Health-related quality of life is a multidimensional construct that reflects an individual’s perception of how their health condition impacts their current level of functioning and satisfaction [12,13], however it may be modulated by factors such as anxiety about interacting with others and difficulty in regulating one’s emotions. For instance, in a recent study on dermatologic patients with hair loss conditions, we found that health-related quality of life did not depend on disease severity clinically defined by the dermatologist as much as on patients’ characterization in terms of anxiety-related traits [14]. Research has only recently started to examine the involvement of emotion regulation mechanisms in psoriasis [5,6] and the issue of whether and to what extent individual differences, specifically those related to difficulty in regulating one’s emotions and anxiety about social interactions, may affect quality of life in psoriatic patients has not received enough attention. A deeper understanding of how HrQoL relates to the clinical and individual characteristics of patients is essential for developing efficient approaches to care for persons with this condition. In the present study, we examine the contribution of emotion dysregulation and social anxiety traits to the impact of psoriasis on HrQoL of affected patients.

## 2. Materials and Methods

A single-center, cross-sectional study was conducted from April to September 2019. Participants were recruited from the Dermatology Unit of Sant’Orsola-Malpighi Hospital, Bologna, as they consecutively approached the outpatient clinic for routine visits. Inclusion criteria were: (i) age > 18, (ii) clinical diagnosis of psoriasis and (iii) absence of comorbid health or neurological conditions. Following dermatological consultation, all patients meeting the inclusion criteria were invited to participate in the study. Approval for the study was obtained by the ethics committee of Sant’Orsola-Malpighi Hospital, Bologna (Prot. Nr. 465/2019, date 13 February 2019) and all patients provided signed informed consent. 

Demographic and clinical information was collected through a paper and pencil self-report questionnaire administered in the clinic. Severity of psoriasis was measured by the same clinician based on the Body Surface Area (BSA) and the Psoriasis Area and Severity Index (PASI) [15]. Patient-reported measures included the following: the Dermatology Life Quality Index (DLQI) [16], which measured the impact of psoriasis on a patient’s quality of life through 10 items (e.g., “How much has your skin condition influenced your work and your study?”); the Psoriasis Skin Appearance Bothersomeness scale (PSAB) [17], which was used to measure patients’ perception of illness severity through three items asking patients to indicate how bothered they were by skin appearance (redness/discoloration), areas of thickness, and scaling/flaking; the Difficulties in Emotion Regulation Scale (DERS) [18], which assessed emotion regulation difficulties in goal-directed behavior (Goals), impulse control (Impulse), acceptance of emotional responses (Nonacceptance), awareness of emotions (Awareness), emotional clarity (Clarity), and access to strategies for regulation (Strategies); and the Social Interaction Anxiety Scale (SIAS) [19], which measured anxiety about social interactions. Items included “I tense up if I meet an acquaintance in the street”.

### Statistical Analysis

We first conducted descriptive statistics and ANOVAs to characterize the sample and test for differences in terms of gender, age, illness severity, lesion site and treatment type. Associations between clinical variables and patient reported measures were assessed by means of Pearson’s r correlation coefficients. We then performed a multiple discriminant analysis (stepwise method) to identify the individual traits that can contribute to discriminating patients reporting no/low impact of psoriasis on quality of life (DLQI < 5; High HrQoL) from those reporting moderate (6 < DLQI < 10) to high (DLQI > 10) impact on quality of life (Low HRQoL). Wilk’s lambda was used as the criterion with univariate F tests for the significance of the extracted function to predict group membership from patient traits. Analyses were conducted with SPSS v.24 with statistical significance set at a 2-sided *p* < 0.05.

## 3. Results

Patient characteristics are represented in Table 1. Significant gender differences were evidenced in terms of age [F1,129 = 18.5; *p* < 0.001], illness age onset [F1,129 = 6.14; *p* = 0.015] and subjectively perceived illness severity (PSAB) [F1,129 = 8.55; *p* = 0.004]. Female patients were younger, had an earlier age of onset of psoriasis and reported higher levels of perceived illness severity compared to males. 

The majority (85%) of our sample was affected by mild psoriasis. Lesions concerned the trunk and upper and lower limbs in 45–50% of the cases and the head in 23% of the cases. Despite the predominance of mild psoriasis in our sample, only half of the patients (49%) reported a relatively good HrQoL. ANOVAs performed to evaluate differences between mild and moderate-to-severe psoriatic patients showed less affected Body Surface Area (BSA) (head [F1,129 = 7.83; *p* = 0.008]; torso [F1,129 = 3.32; *p* < 0.001] and lower extremities [F1,129 = 4.13; *p* = 0.044]) and a better quality of life [F1,129 = 11.86; *p* = 0.001] in mildly affected patients. In addition, patients with more lesions in the head and neck area reported a higher impact of psoriasis on quality of life [F1,129 = 19.5; *p* < 0.001] after controlling for illness severity. 

Patients affected by mild psoriasis did not differ in terms of emotion dysregulation traits [F2,128 = 1.71; *p* = 0.19] and social anxiety [F2,128 = 0.18; *p* = 0.66] from patients affected by moderate-to-severe psoriasis. Among the group of patients affected by mild psoriasis, emotion dysregulation and social anxiety were strongly and positively associated with health-related quality of life (measured by DLQI) as well as with subjectively perceived illness severity (PSAB). Only the awareness of emotions subscale showed a significant relationship with the clinically defined illness severity index (BSA/PASI). Significant correlations were evidenced between emotion dysregulation traits and lesions located in the head and neck area, as well as on lower limbs (see Table 2). 

Considering that our sample was predominantly composed of patients affected by mild psoriasis (110/130), and in order to further investigate whether and to what extent emotion dysregulation traits and social anxiety could contribute to discriminate patients reporting low vs. high HrQoL on top of illness severity, we conducted a multiple discriminant analysis (stepwise method) on patients with mild psoriasis.

A discriminant function was extracted to distinguish the high vs. low HrQoL groups: the Wilks’s lambda test [Wilks = 0.54] and the canonical correlations for the function were highly significant (*p* < 0.001). A high rate of correct classifications was observed for both the high HrQoL (37/44; 84%) and low HrQoL groups (44/53; 88.7%). Overall, the classification matrix showed that a substantial proportion of cases (83.5%) were classified correctly (hit ratio) within each respective group. As revealed by univariate F tests, emotional dysregulation traits and social anxiety contributed significantly to discriminating patients with high vs. low HRQoL (see Table 3). Of all aspects of emotion dysregulation, only lack of emotional awareness did not show significant differences. Lastly, significant differences emerged between the low and high HRQoL groups in terms of gender, with a slightly higher percentage of females (53.2%) than males (46.8%) in the low HRQoL group (*p* = 0.04). 

Student’s *t*-tests performed to compare scores obtained by patients with mild psoriasis in our study with normative data from general population samples in Italy on DERS [20] and SIAS [21] showed that mild psoriasis patients reported significantly higher levels of emotional dysregulation and social anxiety than the normal population samples. Furthermore, the general population’s scores for both DERS and SIAS traits were significantly lower than those reported by mild psoriasis patients reporting a low quality of life.

## 4. Discussion

Patients in our study were predominantly affected by mild psoriasis (85%), which reflects general incidence rates of mild levels of disease affecting the general population in Italy [22]. Moderate-to-severe psoriasis was associated with lower levels of quality of life. However, significantly lower levels of quality of life were also reported by patients with mild psoriasis who were characterized by emotion dysregulation and social anxiety traits. Compared to normal population samples, mild psoriasis patients reported significantly higher levels of both DERS and SIAS traits. These scores were even higher amongst patients who reported low health-related quality of life. Compared to the high HrQoL group of patients, those reporting a low quality of life also showed greater difficulty in accepting emotional responses, engaging in goal-directed behavior and controlling impulses when experiencing negative emotions, they lacked emotion regulation strategies and emotional clarity. The significant correlations of emotion dysregulation traits with specific body parts affected by psoriasis markers such as head and lower limbs are in line with research showing these areas to be particularly sensitive and associated with higher distress [8]. Of all emotion dysregulation facets, only lack of emotional awareness was correlated with the illness severity index (PASI), though it did not yield significant differences between patients with high vs. low HrQoL. This may relate to the fact that psoriasis patients are unusually aware of their own and others’ emotions, which also contributes to high levels of anxiety, especially in social circumstances [5,6]. Our findings also suggest high levels of social anxiety to be a relevant factor in determining low levels of HrQoL. 

These results are in line with research suggesting a high psychosocial burden associated with psoriasis independently from illness severity [1,2,3]. They also highlight the contribution of individual traits to psychosocial burden. While previous research [10] has demonstrated that fear of negative evaluations by others, a core dimension of social interaction anxiety, was strongest among severely affected psoriasis patients, our study provides an even more convincing case for the role of emotion dysregulation and social anxiety traits in discriminating high vs. low HrQoL by focusing on patients affected by mild levels of disease severity. We concede it is possible that emotion dysregulation and social anxiety may be consequential rather than antecedent to a chronic skin condition like psoriasis and suggest that future studies employing longitudinal and case-control designs should more clearly disentangle the contribution of illness-triggered emotion dysregulation and social anxiety in determining quality of life in psoriasis-affected patients. 

In addition, confirming previous research showing that women tend to suffer more intensely from skin diseases [14], higher levels of subjectively perceived illness severity were reported by females compared to males in our sample and a higher percentage of females was found in the low vs. high HrQoL group of patients with mild psoriasis. Given the strong interaction between factors such as female gender, anxiety and emotional dysregulation in chronic skin conditions [14,23,24], future studies should also assess their independent contribution to health-related quality of life in patients affected by psoriasis. 

## 5. Conclusions

The present findings are important in highlighting that individual differences in emotion regulation and social anxiety dimensions should be taken into account as key factors in determining the impact of psoriasis on patient health-related quality of life beyond severity of illness. Important practical implications can be drawn in terms of adopting therapeutic approaches that combine dermatological care with psychosocial approaches, especially approaches focused on enhancing mindfulness-based emotional regulation skills, which are shown to improve clinical outcomes in psoriasis-affected patients [25]. 

## Figures and Tables

**Table 1:** Patient characteristics for the entire sample and for male and female patients separately.

Patient Characteristics	Total (*n* = 130)	Female (*n* = 58)	Male (*n* = 72)
		N (%), Mean (SD)	
Age	46 (14.1)	42.38 (13.8)	48.93 (13.5)
Age onset	25.83 (13.8)	22.41 (2.2)	28.48 (14.4)
Years diagnosed	20.40 (11.8)	20.52 (13.1)	20.30 (10.8)
Body Surface Area (BSA)			
Head	30 (23.1)	13 (43.3)	17 (56.7)
Torso	61 (46.9)	25 (41.0)	36 (59.0)
Upper limbs	64 (49.2)	25 (39.1)	39 (60.9)
Lower limbs	54 (41.5)	32 (59.3)	32 (40.7)
Psoriasis in family	52 (40.0)	26 (50.0)	26 (50.0)
Arthropathic psoriasis	23 (17.6)	12 (52.2)	11 (47.8)
Actual treatment			
Topic therapy	81 (62.3)	40 (49.4)	41 (50.6)
Foto therapy	5 (3.8)	1 (20.0)	4 (80.0)
Traditional therapy	25 (19.2)	11 (44.0)	14 (56.0)
Biological therapy	84 (64.6)	34 (40.5)	50 (59.5)
Illness severity (PASI) *	5.51 (6.1)	6.41 (6.7)	4.79 (2.9)
Mild (<10)	110 (84.6)	47 (42.7)	63 (57.3)
Moderate (10–20)	17 (13.1)	8 (47.1)	9 (52.9)
High (>20)	3 (2.3)	1 (33.3)	2 (66.6)
Subjectively perceived Illness Severity (PSAB)	21.1 (8.4)	23.5 (7.3)	19.1 (8.7)
Dermatologic Life Quality Index (DLQI)	9.39 (10.8)	11.07 (10.6)	8.09 (10.9)
Low (<5)	64 (49.2)	22 (34.4)	42 (65.6)
Moderate (6–10)	17 (13.1)	10 (58.8)	7 (41.2)
High (>10 < 20)	18 (13.8)	9 (50.0)	9 (50.0)
Very high (>20)	27 (20.7)	14 (51.9)	13 (48.1)
Social anxiety (SIAS)	21.50 (10.4)	22.92 (11.1)	20.04 (9.7)
Difficulties in Emotion Regulation Scale (DERS)	84.69 (22.3)	86.37 (21.8)	82.95 (22.5)

Abbreviations: PASI: Psoriasis Area and Severity Index; PSAB: Psoriasis Skin Appearance Bothersomeness scale; DLQI: Dermatology Life Quality Index; SIAS: Social Interaction Anxiety Scale; DERS: Difficulties in Emotion Regulation Scale. * Severity of psoriasis was classified according to clinical diagnostic criteria of the European Medicines Agency (EMA) definitions of mild (PASI < 10), moderate (10 < PASI < 20) and severe psoriasis (PASI > 20).

**Table 2:** Correlations between clinical variables with emotional dysregulation traits and social anxiety in patients with mild psoriasis (*n* = 110).

	PASI	BSA Head	BSA Torso	BSA Upper Limbs	BSA Lower Limbs	PSAB	DLQI
Difficulties in Emotion Regulation Scale (DERS)	0.02	0.278 **	0.028	00.037	0.157	0.31 **	0.46 **
Nonacceptance (DERS)	0.07	0.091	0.000	0.095	0.168	0.34 **	0.41 **
Goals (DERS)	−0.01	0.071	−0.080	0.037	0.210 *	0.29 **	0.33 **
Awareness (DERS)	0.23 *	0.196 *	0.078	−0.155	−0.151	−0.18	0.06
Strategies (DERS)	0.04	0.343 **	0.033	0.086	0.200 *	0.39 **	0.47 **
Clarity (DERS)	0.07	0.215 *	0.023	−0.027	−0.016	0.13	0.27 **
Impulse (DERS)	0.03	0.194 *	0.090	0.064	0.189 *	0.27 **	0.42 **
Social Anxiety (SIAS)	0.00	0.034	0.033	−0.044	0.026	0.25 *	0.29 **

* The correlation is significant at level 0.05 (2 tails). ** The correlation is significant at level 0.01 (2 tails). Abbreviations: PASI: Psoriasis Area and Severity Index; BSA: Body Surface Area; PSAB: Psoriasis Skin Appearance Bothersomeness scale; DLQI: Dermatology Life Quality Index; DERS: Difficulties in Emotion Regulation Scale; Nonacceptance: Difficulties in accepting emotional responses; Goals: Difficulty in engaging in goal-directed behavior; Awareness: Lack of emotional awareness; Strategies: lack of emotion regulation strategies; Clarity: Lack of emotional clarity; Impulse: Difficulty in controlling impulses when experiencing negative emotions; SIAS: Social Interaction Anxiety Scale. Note: Significant correlations are shown in bold.

**Table 3:** Mean (SD) scores reported by general population samples and by patients affected by mild psoriasis in our study reporting high and low levels of health-related quality of life (HrQoL).

	Normative Values	Total Sample (*n* = 110)	High HRQoL (*n* = 53)	Low HRQoL (*n* = 44)
	Mean (SD)	Mean (SD)	Mean (SD)	Mean (SD)
Difficulties in Emotion Regulation Scale (DERS)	73.2 (17.0) ^ab^	84.5 (22.2) ^a^	73.23 (17.4) ^c^	96.5 (21.0) ^bc^
Nonacceptance (DERS)	12.1 (4.4) ^ab^	14.2 (5.2) ^a^	12.1 (4.4) ^c^	16.5 (5.0) ^bc^
Goals (DERS)	12.4 (4.0) ^ab^	12.6 (4.5)	11.1 (3.7) ^ac^	14.6 (4.7) ^abc^
Awareness (DERS)	13.7 (3.6) ^abc^	16.3 (5.0) ^a^	15.7 (5.3) ^b^	16.5 (4.9) ^c^
Strategies (DERS)	14.6 (5.1) ^abc^	17.4 (7.1) ^a^	13.1 (4.5) ^bd^	21.6 (7.9) ^cd^
Clarity (DERS)	9.1 (3.0) ^abc^	11.4 (3.8) ^a^	10.1 (3.4) ^bd^	12.5 (3.8) ^cd^
Impulse (DERS)	11.1 (3.9) ^ab^	12.5 (4.9) ^a^	10.5 (3.4) ^c^	14.9 (5.1) ^bc^
Social Anxiety (SIAS)	17.0 (8.2) ^ab^	21.3 (10.4) ^a^	18.7 (9.1) ^c^	24.5 (11.4) ^bc^

Abbreviations: DERS: Difficulties in Emotion Regulation Scale; Nonacceptance: Difficulties in accepting emotional responses; Goals: Difficulty in engaging in goal-directed behavior; Awareness: Lack of emotional awareness; Strategies: lack of emotion regulation strategies; Clarity: Lack of emotional clarity; Impulse: Difficulty in controlling impulses when experiencing negative emotions; SIAS: Social Interaction Anxiety Scale. Letters a, b, c, d indicate significant comparisons between mean values across groups at *p* < 0.05.

## Data Availability

Data supporting this article can be provided by authors upon reasonable request.
